# Physicochemical Characterization of Hydroxyapatite Hybrids with Meloxicam for Dissolution Rate Improvement

**DOI:** 10.3390/molecules29112419

**Published:** 2024-05-21

**Authors:** Lauretta Maggi, Valeria Friuli, Beatrice Cerea, Giovanna Bruni, Vittorio Berbenni, Marcella Bini

**Affiliations:** 1 Department of Drug Sciences, University of Pavia, Viale Taramelli 12, 27100 Pavia, Italy; lauretta.maggi@unipv.it; 2 Department of Information Engineering, University of Brescia, Via Branze 38, 25123 Brescia, Italy; beatrice.cerea@unibs.it; 3 Department of Chemistry, University of Pavia, Viale Taramelli 16, 27100 Pavia, Italy; giovanna.bruni@unipv.it (G.B.); vittorio.berbenni@unipv.it (V.B.); 4 Consorzio per i Sistemi a Grande Interfase (CSGI), Department of Chemistry, University of Pavia, Viale Taramelli 16, 27100 Pavia, Italy; 5 National Reference Centre for Electrochemical Energy Storage (GISEL), Consorzio Interuniversitario Nazionale per la Scienza e Tecnologia dei Materiali (INSTM), Via G. Giusti 9, 50121 Firenze, Italy

**Keywords:** meloxicam, drug delivery, in vitro dissolution studies, hydroxyapatite, hybrids

## Abstract

Organic–inorganic hybrids represent a good solution to improve the solubility and dissolution rates of poorly soluble drugs whose number has been increasing in the last few years. One of the most diffused inorganic matrices is hydroxyapatite (HAP), which is a biocompatible and osteoconductive material. However, the understanding of the hybrids’ functioning mechanisms is in many cases limited; thus, thorough physicochemical characterizations are needed. In the present paper, we prepared hybrids of pure and Mg-doped hydroxyapatite with meloxicam, a drug pertaining to the Biopharmaceutical Classification System (BCS) class II, i.e., drugs with low solubility and high permeability. The hybrids’ formation was demonstrated by FT-IR, which suggested electrostatic interactions between HAP and drug. The substitution of Mg in the HAP structure mainly produced a structural disorder and a reduction in crystallite sizes. The surface area of HAP increased after Mg doping from 82 to 103 m^2^g^−1^ as well as the pore volume, justifying the slightly high drug amount adsorbed by the Mg hybrid. Notwithstanding the low drug loading on the hybrids, the solubility, dissolution profiles and wettability markedly improved with respect to the drug alone, particularly for the Mg doped one, which was probably due to the main distribution of the drug on the HAP surface.

## 1. Introduction

The inorganic materials (such as hydroxyapatites, silica or metals) emerged in the last few last years as interesting support for the production of drug delivery systems (DDSs) [1,2], and they are particularly useful for poorly water-soluble drugs, which are widely diffused in the pharmaceutical market [3,4]. The fate of a drug in solid form (tablets, capsules, or suspensions) after oral administration and before adsorption is its release and dissolution in the gastrointestinal fluids [5,6]. For many poorly water-soluble drugs of the Biopharmaceutical Classification System (BCS) class II, the dissolution rate is the limiting step in their bioavailability, and this parameter is in turn related to the surface area available for dissolution. The low solubility that characterizes these drugs represents a problem during the formulation process and can explain the differences in oral bioavailability.

The rate and completeness of the adsorption of problematic drugs can be improved by many strategies, such as particle size reduction, use of solid dispersions or salts, micelles, liposomes or organic–inorganic hybrids [7,8,9]. These last systems possess many intriguing features that allow being applied as carriers for controlled and/or targeted drug release as well as to improve the long-term stability and storage of drugs by protecting them from unsafe environments [10,11,12]. Hydroxyapatite (Ca_10_(PO_4_)_6_ (OH)_2_) (HAP) is often the preferred inorganic support for DDS thanks to its biocompatibility, bioactivity, osteoconductivity and ease in bonding to living tissues and to molecules through simple adsorption [13,14]. It can be also easily synthesized with many different synthetic routes, obtaining different morphological features, as well as easily doped with cations substituting Ca and/or P ions [15,16].

Poorly water-soluble drugs can pertain to different classes of the BCS; meloxicam pertains to class II, i.e., drugs with low solubility and high permeability. Thanks to these features, it can be used as a model drug to test the efficiency of hybrids based on hydroxyapatites to improve its pharmaceutic characteristics.

Meloxicam (Mlx, 4-Hydroxy-2-methyl-N-(5-methyl-2-thiazolyl)-2H-1,2-benzothiazine-3-carboxamide-1,1-dioxide, C_14_H_13_N_3_O_4_S_2_) is a non-steroidal anti-inflammatory drug (NSAID) of the oxicam class with analgesic and antipyretic properties. It can be used to treat arthritis and osteoarthrosis for a limited time and rheumatoid arthritis for longer times [17]. From the structural point of view, Mlx has five polymorphic forms [18], but only Form I is commercialized [19]. Meloxicam is insoluble in water, slightly soluble in methanol and ethanol, and highly soluble in DMF and DMSO [20] and its pKa is 4.08 [21].

The hydroxyapatite in DDS should bond to drugs by a simple adsorption process, but in many cases, the interaction between the involved entities has not been analyzed in detail and was taken for granted. To this aim, a thorough physicochemical characterization of hydroxyapatite is first needed. Its structure, morphology, surface area, spectroscopic features and chemical composition should be measured. By analyzing the same characteristics on the hybrids, it will be possible to investigate the relationships between the physicochemical properties, the drug loading and the subsequent dissolution behavior.

The aim of the paper was to synthesize hybrids of meloxicam with pure and Mg-doped HAP inorganic hosts. A complete physicochemical study was performed to first characterize the hydroxyapatites and then prove the hybrids formation. X-Ray Powder Diffraction (XRD) with Rietveld refinement, Fourier Transform infrared spectroscopy (FT-IR), Differential Scanning Calorimetry (DSC) and Scanning Electron Microscopy with Energy-Dispersive Spectroscopy (SEM-EDS) were applied together with adsorption measurements to determine the specific surface area and the pore volume and distribution of hydroxyapatites to be possibly related to the pharmaceutical results. In vitro dissolution tests along with solubility and contact angle measurements in different media that simulate the gastrointestinal environments allowed us to test the suitability of the hybrids as drug delivery systems.

## 2. Results

### 2.1. Physicochemical Characterization

#### 2.1.1. XRD and Rietveld Results

**Figure 1:**
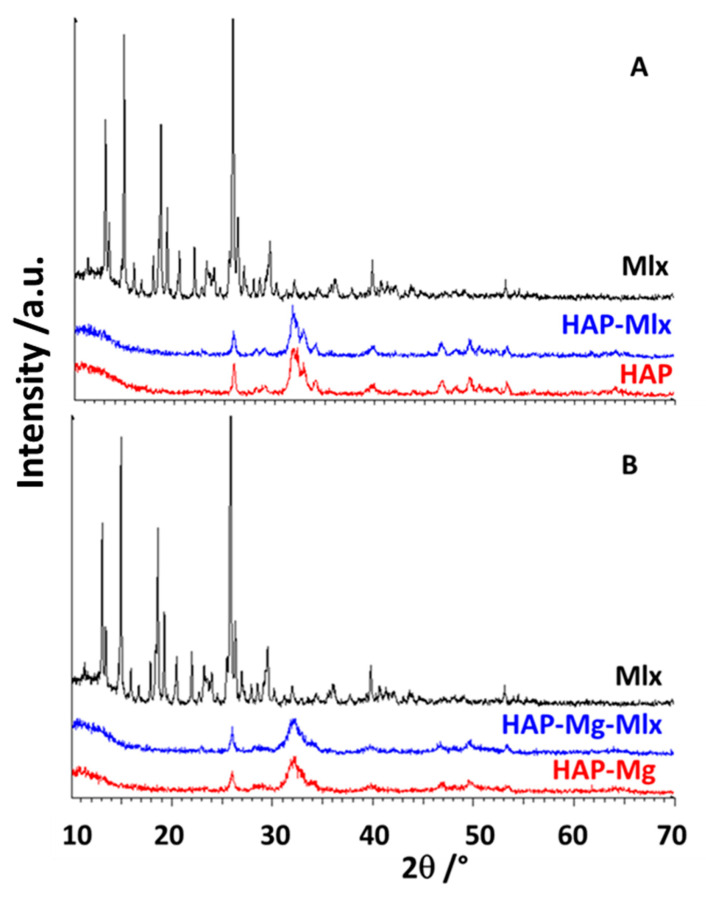
XRD patterns of (**A**) HAP and HAP-Mlx and (**B**) HAP-Mg and HAP-Mg-Mlx compared with the pattern of pure meloxicam.

The XRD patterns of Mlx, HAP and HAP-Mlx are compared in Figure 1A, and those of Mlx, HAP-Mg and HAP-Mg-Mlx are compared in Figure 1B.

The pattern of Mlx shows well-defined peaks typical of a crystalline drug that possesses the polymorphic Form I [9,12]. The pure hydroxyapatite obtained from the co-precipitation method (Figure 1A) showed the typical pattern of the hexagonal structure with the main peaks in the 30 and 35° 2θ angular range [22]. The pattern of the HAP-Mlx hybrid resembles that of HAP; no peaks due to Mlx are evident. The pattern of the HAP-Mg sample (Figure 1B) is like that of HAP apart from an evident broadening of the peaks. The HAP-Mg-Mlx pattern is practically superimposable to that of HAP-Mg and no peaks of the drug are evident, similarly to HAP-Mlx. These results can suggest that the drug is present in the hybrids in an amorphous state. To verify this hypothesis, XRD patterns of physical mixtures of HAP and HAP-Mg with Mlx (in the ratio obtained from the pharmaceutical study, see Section 2.2.1) were collected (Figure S1). The main peaks of Mlx are clearly seen in the patterns (see the cross in the figure) with a low intensity due to its low amount.

**Figure 2:**
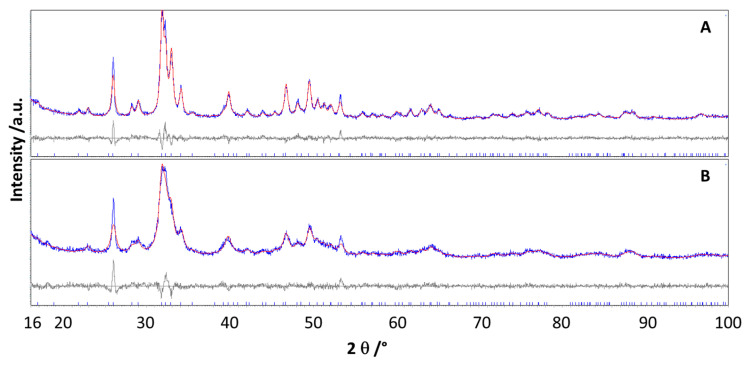
Rietveld refinement results of HAP-Mlx (**A**) and HAP-Mg-Mlx (**B**) sample patterns. The experimental patterns (blue) are compared with the calculated ones (red). In the bottom part, the difference curve (gray) and the bars of the expected peak positions are also shown.

The Rietveld structural refinement was performed on HAPs and on the hybrid’s patterns on the basis of the crystallographic model of hydroxyapatite. In fact, due to the absence of the drug peaks and the similarity of the patterns, we can suppose that the hybrids maintain the structural features of the main hydroxyapatite phase. The HAP crystal structure is hexagonal (*P6_3_*/*m* space group) with Ca ions located on two distinct crystallographic sites: Ca1 with a ninefold coordination and Ca2 with a sevenfold coordination [23]. The detailed patterns, expressly collected to perform the Rietveld refinement, are compared in Figure S2. The main refined structural parameters are reported in Table 1 together with the bond lengths values derived from the refined crystallographic positions and lattice parameters. As an example, the comparison between HAP-Mlx and HAP-Mg-Mlx experimental patterns and the calculated ones is shown in Figure 2, while the other comparisons are reported in Figures S3 and S4.

All the refinements are reliable, as suggested by the agreement indices (Table 1), as well as by the graphical comparisons in Figure 2, Figures S3 and S4.

The lattice parameters are in line with those of other hydroxyapatites [23,24]. The Mg-doped sample has the same value for the lattice parameter *a* of pure HAP, while the *c* value is slightly lower. The hybrids have the same lattice parameters of the corresponding HAPs alone. The crystallite sizes of HAP and HAP-Mlx are about 23 nm, while for HAP-Mg and HAP-Mg-Mlx they are almost halved, which is in line with the observation of XRD peaks broadening (Figure 1).

The atomic positions are slightly different between the samples, but a particular trend cannot be evidenced. Mg dopant, in principle, could occupy both the calcium sites. So, two different crystallographic models (Mg on Ca1 or Ca2 site, occupation values of 0.05 and 0.95 for the 5% Mg amount, respectively) were tried by allowing the occupations to vary. For the model with Mg on the Ca2 site, we can find values of 0.042(7) and 0.958(7), while when Mg was located on the Ca1 site, its occupation was practically near zero. So, from the obtained occupation values and from the agreement indices, it was determined that the most correct model is that with Mg ions on Ca2 sites. The mean bond lengths determined for the Ca2 site (Table 1) do not show a peculiar trend neither with Mg doping nor with the drug. The case of the Ca1 and P sites is different. The mean bond lengths of the Ca1 site tend to increase passing from HAP to HAP-Mg, and a further increase seems evident for HAP-Mg-Mlx. For the P site instead, a decrease of the mean bond length was verified for Mg-doped samples with respect to the HAPs.

#### 2.1.2. Thermal Analysis

**Figure 3:**
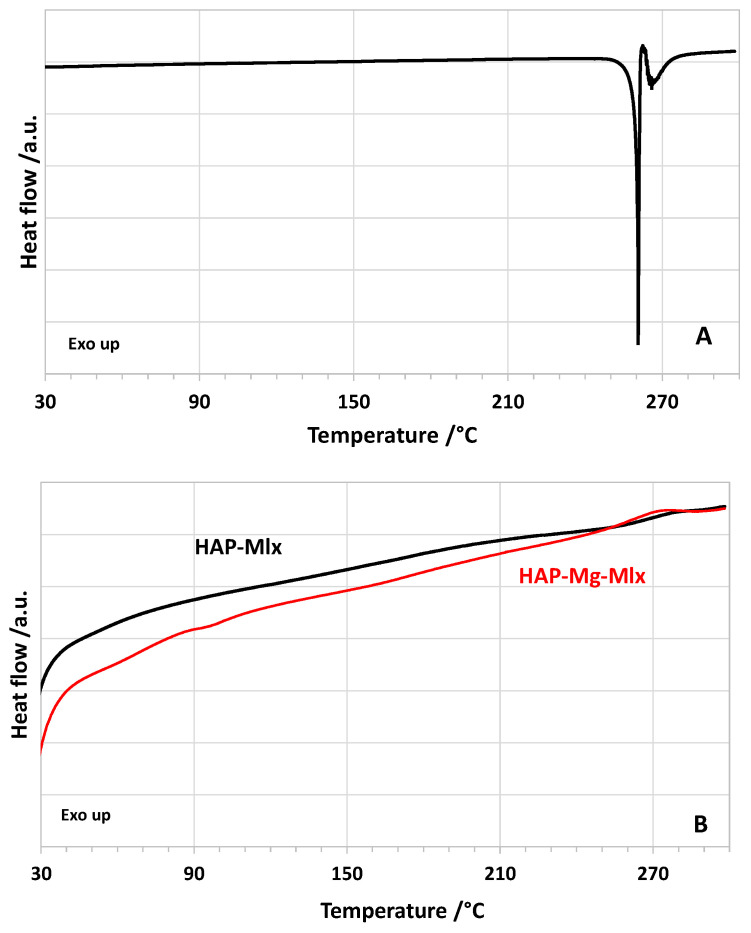
DSC curves of (**A**) Mlx and (**B**) HAP-Mlx and HAP-Mg-Mlx samples. In the figures, the exothermic transitions are oriented upward.

The DSC curves of meloxicam and hybrids are reported in Figure 3A,B, respectively. Mlx shows an endothermic peak of melting at about 265 °C, which was followed by a rapid decomposition in agreement with the thermal behavior of the Form I polymorph [9]. The two hybrids (Figure 3B) show instead flat DSC curves without relevant thermal events. The melting peak of the drug is not evident, suggesting its amorphous nature.

#### 2.1.3. Spectroscopic Analysis

**Scheme 1:**
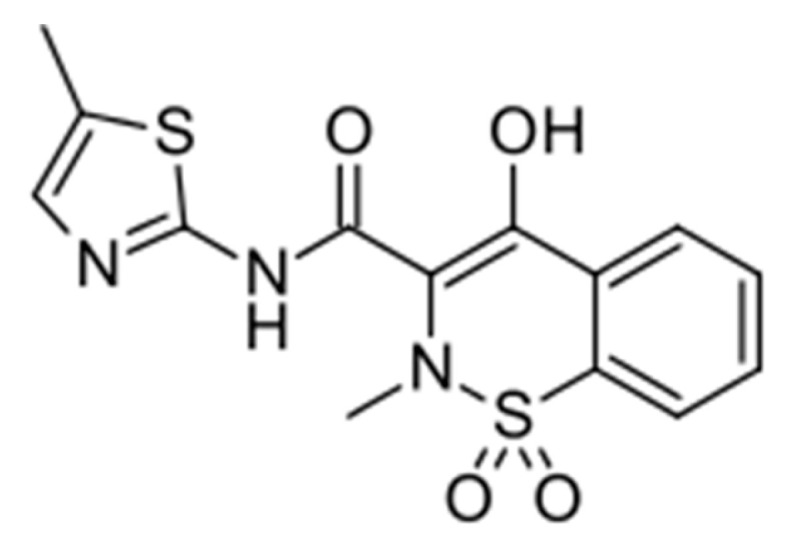
Molecular structure of meloxicam.

**Figure 4:**
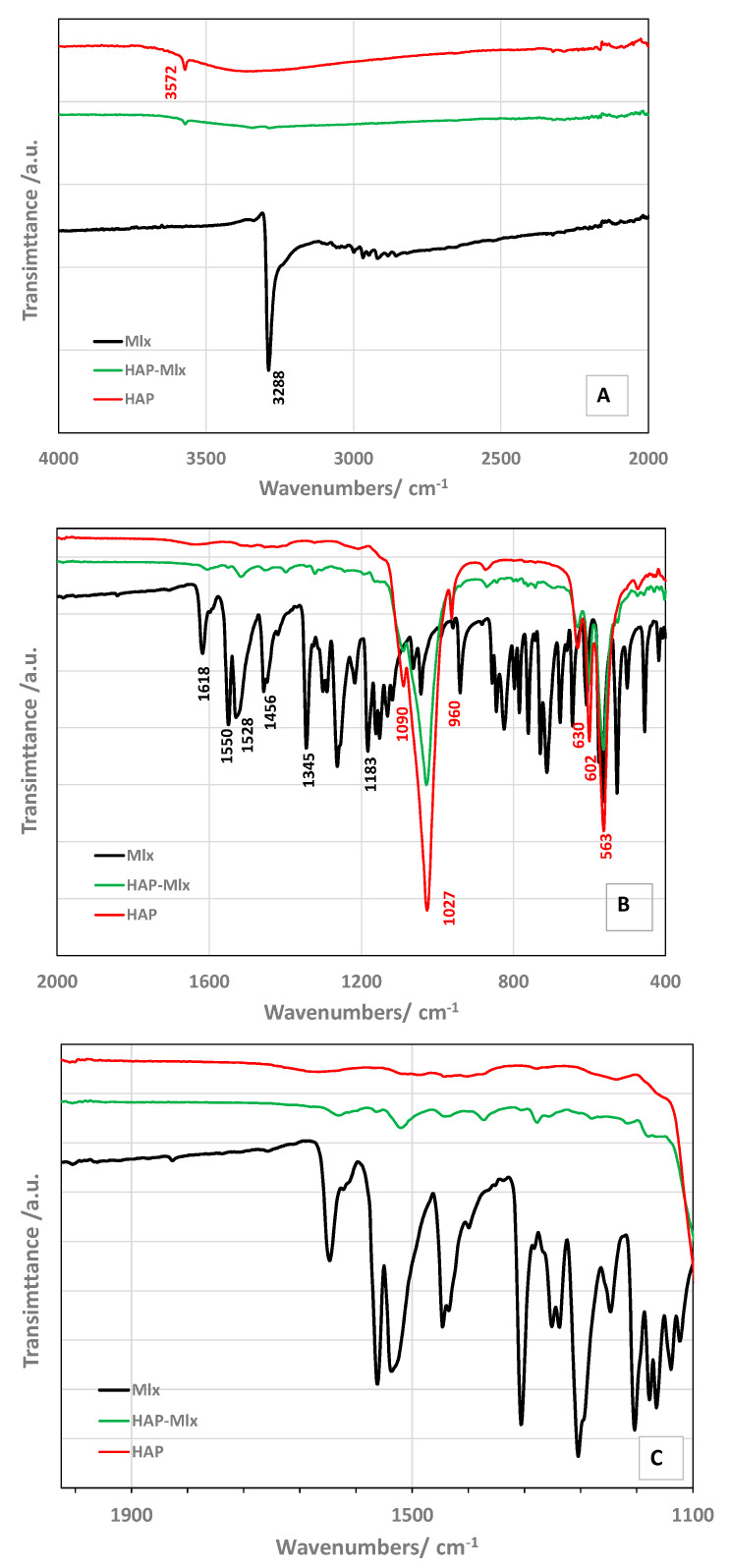
FT-IR spectra of Mlx, HAP and HAP-Mlx in the spectral ranges 4000–2000 cm^−1^ (**A**) and 2000–400 cm^−1^ (**B**). In (**C**), a further enlargement of the 2000–1100 cm^−1^ spectral range is provided.

**Figure 5:**
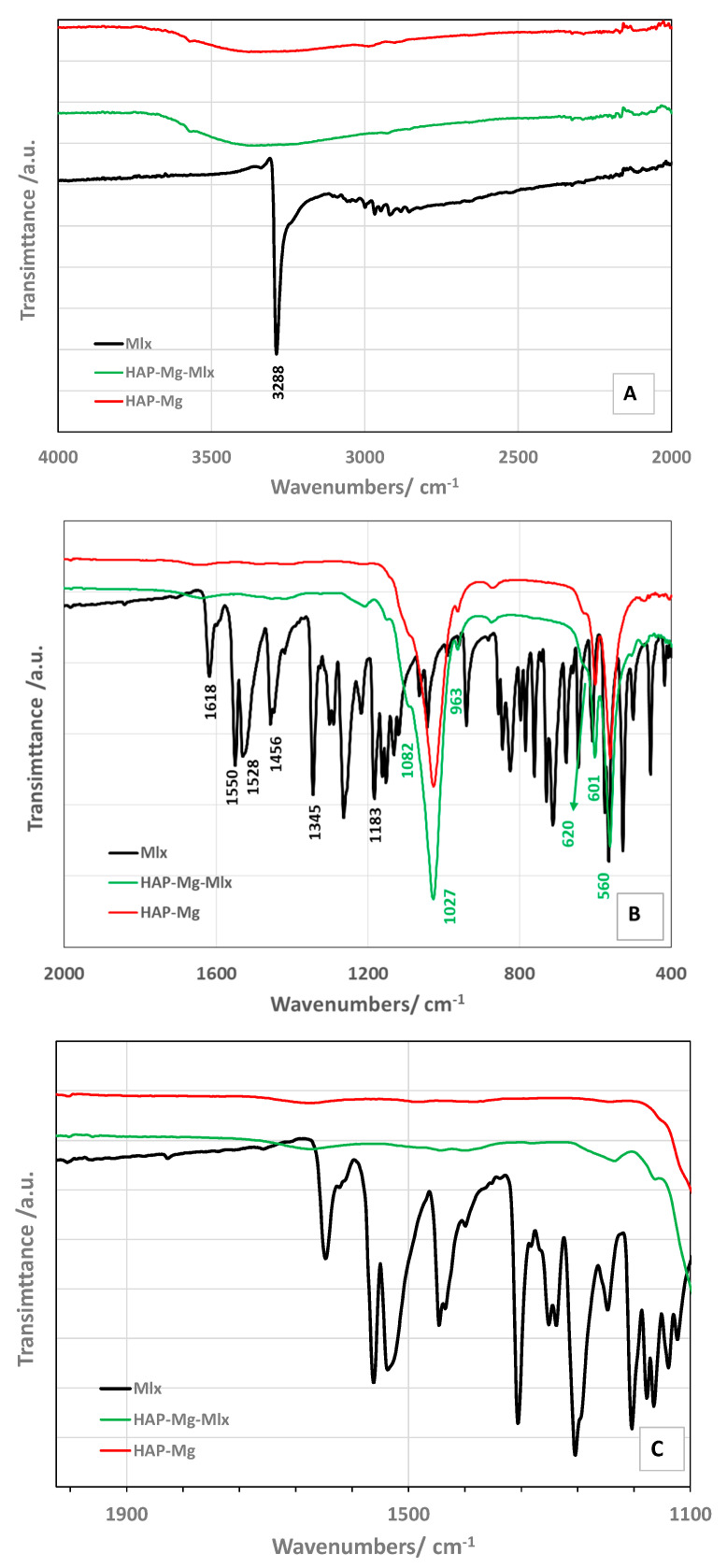
FT-IR spectra of Mlx, HAP-Mg and HAP-Mg-Mlx in the spectral ranges 4000–2000 cm^−1^ (**A**) and 2000–400 cm^−1^ (**B**). In (**C**), a further enlargement of the 2000–1100 cm^−1^ spectral range is provided.

The FT-IR spectroscopic study was performed on meloxicam, HAPs and the hybrids (Figure 4 and Figure 5). The active ingredient has the molecular structure reported in Scheme 1. Its main absorptions are located at 3288 cm^−1^ (NH stretching), 1618 cm^−1^ (stretching of amide C=O), 1550–1528–1456 cm^−1^ (stretching of aromatic ring), and 1345 and 1183 cm^−1^ (asymmetric and symmetric stretching of SO_2_) [9] (Figure 4 and Figure 5).

The pure and Mg-doped hydroxyapatites have similar spectra, and their main adsorptions are due to phosphate vibrational bands: the bending of the O-P-O group is responsible for the peaks at about 560 cm^−1^ and 600 cm^−1^, the band at about 960 cm^−1^ is due to the stretching of the PO_4_^3−^ group, and the asymmetric P-O stretching produces the bands at around 1000 cm^−1^. The peaks at 3572 cm^−1^ and at about 620–630 cm^−1^ are due to O–H stretching and bending, respectively. In the HAP-Mg-doped sample spectrum, however, the peak at 3572 cm^−1^, well evident in the HAP, is very low, and that at 630 cm^−1^ is slightly shifted at a lower wavenumber (620 cm^−1^). It is also obvious that the whole quantum yield of the HAP-Mg spectrum is lower with respect to the pure HAP.

The two hybrids show spectra very similar to those of the corresponding hydroxyapatites. However, by enlarging the 2000–1100 cm^−1^ spectral range (Figure 4C and Figure 5C), weak and broad peaks, corresponding to those of Mlx, are present. This is particularly true for the HAP-Mlx hybrid (see also Figure S5), while for HAP-Mg-Mlx, these weak peaks appear broadened, which is in agreement with the lower intensities of all the peaks of the spectrum. The main peaks of hydroxyapatites and Mlx in the hybrids are shifted by a few wavenumbers with respect to those of the corresponding pure phases, suggesting the formation of weak interactions between them and meloxicam.

#### 2.1.4. Morphological and Compositional Analysis

**Figure 6:**
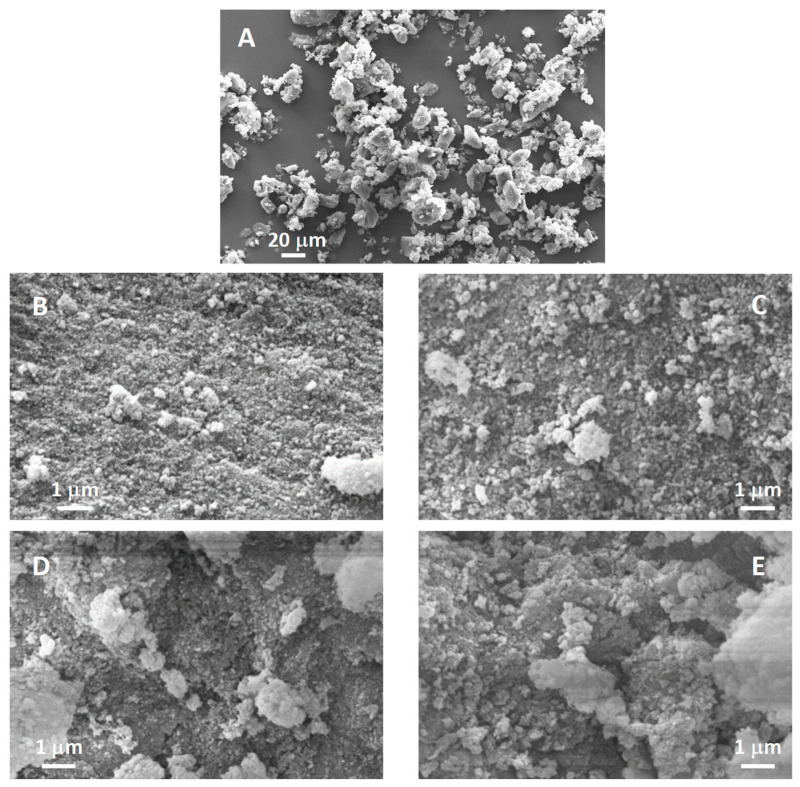
SEM images of (**A**) Mlx, (**B**) HAP, (**C**) HAP-Mg, (**D**) HAP-Mlx and (**E**) HAP-Mg-Mlx samples.

SEM micrographs are reported in Figure 6. The Mlx drug alone (Figure 6A) is constituted by big particles (up to 20 μm). Most of them have an irregular parallelepiped shape. HAP shows aggregates of small spherical particles (Figure 6B) that do not change after Mg doping (Figure 6C). The hybrid’s morphology is like that of the corresponding hydroxyapatite (Figure 6D,E).

The chemical composition of the samples was evaluated by using EDS microanalysis. The EDS spectrum of HAP-Mg sample, chosen as an example, is reported in Figure S6 together with the maps showing the distribution of the different elements. The Ca, Mg and P amounts were analyzed for all the samples and compared with the stoichiometric values: the results are reported in Table 2.

The stoichiometric ratios reported in Table 2 refer to the stoichiometry of the prepared hydroxyapatites (i.e., Ca_10_(PO_4_)_6_(OH)_2_ with a Ca/P ratio of 10/6 = 1.67 and Ca_9.5_Mg_0.5_(PO_4_)_6_(OH)_2_ with a Mg/Ca ratio of 0.5/9.5 = 0.053), while the corresponding observed values come from the amount determined by EDS.

For all the samples, the observed chemical compositions are in good agreement with those expected on basis of the samples’ stoichiometry by also taking into considering the error of EDS determination.

#### 2.1.5. Adsorption Measurements

**Figure 7:**
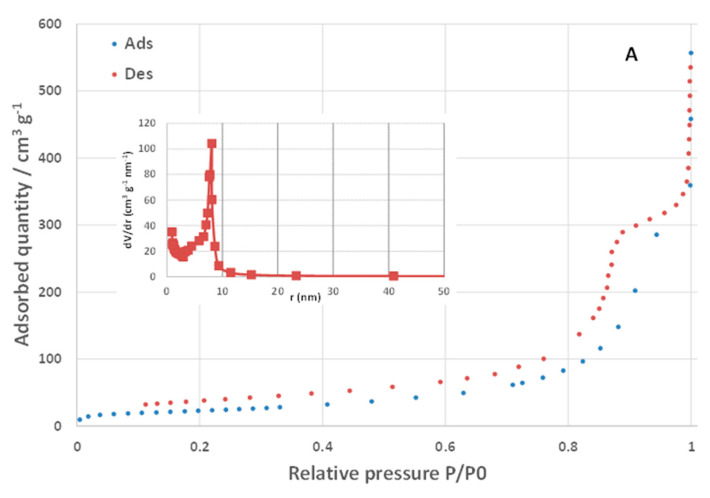

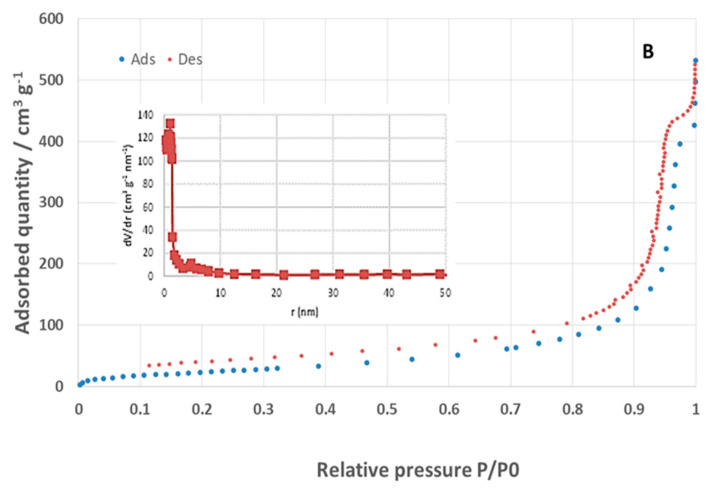
Hysteresis cycle of the adsorption measurements of (**A**) HAP and (**B**) HAP-Mg sample. The pore distribution is also shown in the inset for both the samples.

The N_2_ adsorption/desorption curves of HAP and HAP-Mg are shown in Figure 7A,B, respectively, together with the pore distribution (see inset).

The isotherms of the HAPs samples can be classified as Type IV and, with a more detailed classification referring to the pore forms, to the H1 type. From the linear part of the curves (BET part), a surface area of 82 and 103 m^2^g^−1^ for HAP and HAP-Mg was found. The pore volume was 0.44 cm^3^g^−1^ for HAP and 0.61 cm^3^g^−1^ for HAP-Mg samples, while the pore distribution (see insets) showed a maximum at around 8 nm for HAP (mesoporous) and 1.5 nm for HAP-Mg (microporous) samples.

### 2.2. Pharmaceutical Results

#### 2.2.1. Drug Loading and Solubility

The drug loading (wt%) was determined as 1.20 ± 0.05% for the HAP-Mlx sample and 1.66 ± 0.07% for the HAP-Mg-Mlx sample from UV detection.

The water solubility of both hybrids, 55.8 ± 2.7 mg/L for HAP-Mlx and 26.6 ± 3.4 mg/L for HAP-Mg-Mlx (see Figure S7), was much higher than the solubility of pure meloxicam, 8.0 ± 0.2 mg/L, which was measured under the same conditions [25].

#### 2.2.2. Dissolution Tests

**Figure 8:**
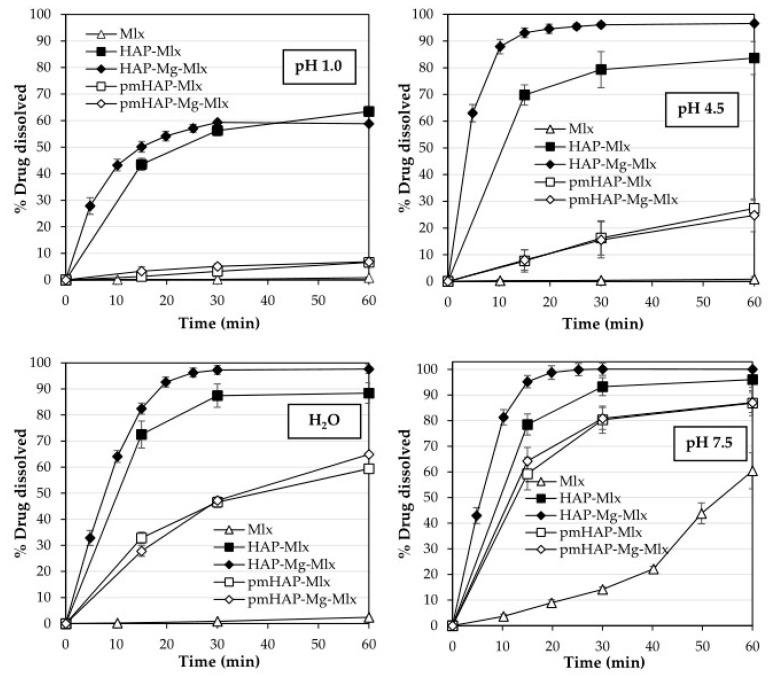
Dissolution profiles of HAP-Mlx and HAP-Mg-Mlx samples compared to the corresponding physical mixtures, pmHAP-Mlx and pmHAP-Mg-Mlx, and pure meloxicam in the different fluids considered. All samples contain 7.5 mg of the drug.

Mlx loading on the two types of HAP significantly increased the dissolution rate of the drug in all four fluids considered (Figure 8). The improvement is evident even in comparison to the corresponding physical mixtures.

In particular, at pH 1.0, a condition that simulates a fasted gastric environment, there is an increase of at least 6 times in the fraction of the dose that passes in solution in 1 h compared to physical mixtures and even more if compared to the pure Mlx, which is practically insoluble in these conditions. Under these conditions, the two HAPs have comparable behavior. Even at pH 4.5, which simulates gastric conditions in the presence of food, a great improvement in the dissolution rate of the hybrids can be evidenced both in comparison to physical mixtures and to Mlx alone. In this case, HAP-Mg-Mlx shows a faster dissolution trend than HAP-Mlx, and it is also able to release the entire dose in 15–20 min. In an unbuffered medium such as deionized water, the behavior of different samples is similar to that at pH 4.5, although as the pH increases, the drug becomes progressively more soluble. The phosphate buffer at pH 7.5 is the fluid prescribed by the US Pharmacopeia monograph for the quality control of oral pharmaceutical forms containing Mlx because it is the only physiological condition (this pH is reached in the most distal portion of the intestine) in which the drug is soluble. At pH 7.5, the drug passes into solution very quickly from both HAP-Mg-Mlx and HAP-Mlx, more slowly but still completely from the physical mixtures pmHAP-Mlx and pm HAP-Mg-Mlx; Mlx dissolves although much more slowly.

#### 2.2.3. Contact Angle

**Figure 9:**
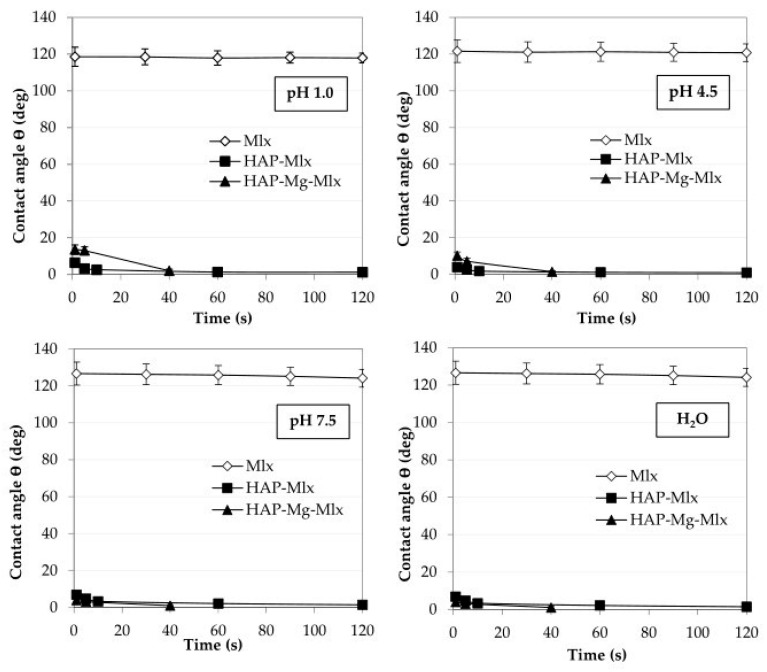
Contact angle, θ, of HAP-Mlx and HAP-Mg-Mlx compared to Mlx alone in the four fluids considered: HCl pH 1.0, phosphate buffers, pH 4.5 and 7.5, and deionized water.

On both the hybrids and on Mlx, the contact angle was also measured (Figure 9). For HAP-Mlx and HAP-Mg-Mlx, the contact angle drops from the initial values between 3° and 10° to zero in just a few seconds, while Mlx is much less wettable and shows a constant value of 120° in all the fluids. The pH or the buffering power of the different fluids seem to have little or no influence on the wettability of the analyzed samples.

## 3. Discussion

The physicochemical characterization of the samples was performed by the combined use of many techniques, providing various features of both pure and doped hydroxyapatites and hybrids. In fact, the employed techniques, based on distinct operating principles, also have different sensitivity and could evidence different features of pure samples and hybrids. In particular, the drug presence was proved by FT-IR spectroscopy, while the dissolution tests were able not only to determine the drug presence but also to provide its quantification. The other characterization techniques cannot provide insights into the Mlx presence, which is mainly due to its amorphous nature and/or to its low amount.

X-ray powder diffraction is a well-known powerful technique that can provide the structural features of materials. First, it was demonstrated, by using XRD measurements with Rietveld refinements, that Mg can easily substitute Ca in the hydroxyapatites structure, at least for the employed amount. The crystal structure does not change after doping, but an evident peaks’ broadening was observed. This can suggest the presence of a structural disorder caused by the dopant introduction that limits the crystallite size growth, as clearly demonstrated by the nearly halved crystallite sizes values of HAP-Mg compared to HAP (Table 1). The introduction of disorder and the effect on crystallite size values were also found for other kinds of doped samples [26]. A contraction of the *c* lattice parameter was determined on the HAP-Mg sample due to the lower ionic radius of Mg^2+^ compared to Ca^2+^ (0.89 Å with respect to 1.12 Å for eightfold coordination). Other differences of HAP-Mg concerning HAP have been obtained on the M-O bond lengths of the cations (Table 1). Mg^2+^ ions, with a high electronegativity compared to Ca^2+^, can form stronger bonds, influencing the bond lengths not only of the Ca2 site where they are located but also of the neighboring P and Ca1 sites, which are connected to them by oxygen atoms. From the XRD results, we supposed that the drug turned out to be in amorphous form, which was due to the absence of its peculiar reflections and that the drug presence on the HAP surface does not affect the structural parameters of the hydroxyapatite itself.

The absence of Mlx melting peak in the DSC curves of the hybrids was another proof of the amorphous state of the active ingredient, which can be considered as a very positive tool because the drug can be more bioavailable with respect to its crystalline form. The reduction in crystallinity of the Mg-doped HAP too could be considered a positive aspect of the obtained DDS, possibly favoring its dissolution rate.

The Mg presence in the hybrids was quantitatively demonstrated by the EDS measurements. In fact, it can be seen that the sample’s stoichiometry agrees with that of the synthesis not only for the Mg amount but also for Ca and P ratios. On the other hand, it is known that the hydroxyapatite structure can sustain different kinds of doping, on both Ca and P sites, also in relatively high amount [15,27].

The Mg dopant, as well as the Mlx presence, does not change the morphology of the hydroxyapatite, which appeared, for all the samples, constituted by agglomerates of spherical particles. The adsorption measurements evidenced interesting features for HAPs. Both pure and Mg-doped HAP showed Type IV isotherms with H1 form that is often associated with porous materials consisting of well-defined cylindrical-like pore channels or agglomerates of approximately uniform spheres. They also presented low-pressure hysteresis, which may be associated with the change in volume of the adsorbent, for example, the swelling of non-rigid pores or the irreversible uptake of molecules in pores with similar width as that of the adsorptive molecule. The surface area and pore volumes were different for the two HAPs: the HAP-Mg has higher values, which could also justify the slightly higher loaded drug amount. The pore distribution was also different: while for HAP, a maximum at about 8 nm was evidenced, for HAP-Mg, the maximum was located at about 1.5 nm. The doping produced changes in the main porosity of the material, passing from mainly mesoporous to microporous, following the IUPAC classification of the pores. These results agree with published results on the physical features of doped hydroxyapatites [27]. This evidence can further justify the slightly higher amount of adsorbed drug on the HAP-Mg sample.

From the IR spectroscopic measurements, a clear proof of the drug’s presence was obtained. The bands due to the active ingredient were weak due to its low concentration and to the high difference in the molar mass. They were also shifted, as well as those of HAPs, with respect to the original positions, suggesting the formation of weak interactions between Mlx and HAP. For HAP-Mg-Mlx, the drug peaks were also broadened, suggesting a stronger interaction with the HAP substrate and a more effective hybrid for the enhanced drug release. We can also try to go into detail regarding the possible interaction between HAP and meloxicam. It is reported that HAP possesses two main adsorption sites, Ca^2+^ and PO_4_^3−^, that can be differently involved in different media with variable pH conditions [28]. In our case, HAPs are dispersed in a drug solution that has a neutral or slightly basic pH. In these conditions, the HAP surface should be able to form basic hydrogen bonds with the species present in the medium (the drug), probably involving the SO_2_ or NH groups of the drug. However, it cannot be excluded that the hydroxyl of the drug could also bond to positively charged Ca when pH is slightly basic. So, electrostatic interactions between meloxicam and HAPs seem very plausible. A possible confirmation of the proposed mechanism could come from the absence of the NH stretching peak typical of Mlx in the FT-IR spectra of the hybrids due to its involvement in the bond with HAP. The same scenario could be valid for HAP-Mg in which the presence of Mg produced a disorder in the structure and an increase in surface area and a decrease in the pore dimensions. It is possible that a higher number of sites could be disposable for adsorption on HAP-Mg, mainly located onto the surface, and thus more favorable to adsorb the drug but also to easily release it.

Both the hybrids with meloxicam possessed improved wettability, as demonstrated by the low values of contact angles compared to the pure drug. This evidence can also explain the increase in the dissolution rate of the hybrids compared to the pure drug. In particular, the HAP-Mg-Mlx showed a faster dissolution trend than HAP-Mlx, and it can release the entire dose in 15–20 min. This represents a huge advantage for a pain-relieving drug like Mlx, which should be available for absorption in the shortest possible time for the onset of a rapid therapeutic effect.

So, we can conclude that the hybrids’ formation has been, in general, very positive for the improvement of the Mlx solubility and dissolution rate. These features are particularly advantageous when Mg dopant is present, suggesting that doped hydroxyapatites can be considered as amazing inorganic matrixes for the preparation of appealing DDS.

## 4. Materials and Methods

### 4.1. Syntheses

Meloxicam (Mlx) was generously donated by Olon (Casaletto Lodigiano, LO, Italy). The other reagents of analytical grade were purchased from Sigma Aldrich (Saint Louis, MO, USA) and used as received.

#### 4.1.1. HAPs Synthesis

For the preparation of pure and Mg-doped hydroxyapatites, the co-precipitation method was chosen. For the preparation of the pure sample Ca_10_(PO_4_)_6_(OH)_2_, stoichiometric amounts (Ca:P ratio 1.67) of Ca(NO_3_)_2_ 4H_2_O and (NH_4_)_2_HPO_4_ have been dissolved separately in 30 mL of water with magnetic stirring. Both solutions were basified with NH_4_OH until a pH value of about 10–11 was obtained. Then, the solution of phosphate was slowly added drop by drop under stirring to that of calcium and the pH was controlled, possibly adding other NH_4_OH. The temperature was then raised to 80 °C and maintained for 1 h. After cooling, the solution was centrifuged to collect the powder (6000 rpm for 5 min) that was washed three times with distilled water and placed in an oven for 22 h at 100 °C. This sample will be named HAP.

To obtain the Mg 5% doped hydroxyapatite (Ca_9.5_Mg_0.5_(PO_4_)_6_ (OH)_2_), calcium nitrate and Mg(NO_3_)_2_ 6H_2_O were dissolved together in 30 mL of water with magnetic stirring, maintaining the (Ca + Mg)/P ratio to 1.67. The synthesis then proceeded as for the pure sample. This sample will be named HAP-Mg.

#### 4.1.2. Hybrids’ Preparation

The drug is poorly water soluble, as discussed, so for the synthesis of hybrids, it has been necessary to find a suitable medium for its solubilization. After many trials, the best-identified medium has been a mixture of 3:1 *v*/*v* ethanol/water.

Meloxicam (100 mg) has been added to 10 mL of the solution of ethanol/water (3:1 ratio) and magnetically stirred until the drug was completely dissolved. Then, 150 mg of HAP or HAP-Mg has been added to the drug solution; the dispersion was sonicated for about 5 min and maintained under stirring at room temperature for 24 h. The dispersions have been centrifuged at 6000 rpm for 5 min, and the collected powders were dried in an oven at 50 °C overnight. The pure and doped hybrids were named HAP-Mlx and HAP-Mg-Mlx, respectively.

### 4.2. Physical–Chemical Characterizations

X-ray powder diffraction (XRPD) measurements were performed by using a Bruker D5005 diffractometer (Bruker BioSpin, Fällanden, Switzerland) with the Cu Kα radiation, graphite monochromator and scintillation detector. The patterns were collected in air with a step size of 0.03° and counting time of 2 s/step in the angular range 10–70° by using a low background silicon sample holder. The structural refinement with the Rietveld method (TOPAS 3.0 software, Bruker BioSpin, Fällanden, Switzerland) was used to determine the main structural parameters of HAPs and hybrids, starting from the crystallographic model of hexagonal HAP. In particular, the background coefficients, the displacement error, the scale factor, the lattice parameters, crystallite sizes, the atomic positions, thermal parameters and occupancies were varied. Use was made of the fundamental parameter function to fit the peaks profile and Scherrer equation to determine the crystallite sizes. Two different structural models were tried with Mg on Ca1 or Ca2 sites to find its more likely position. To this aim, more detailed patterns were collected in the following conditions: 16–100°, 0.03° step size and 15 s/step of counting time.

Fourier-Transformed Infrared (FT-IR) spectra were obtained with a Nicolet FT-IR iS20 spectrometer (Nicolet, Madison, WI, USA) equipped with ATR (Attenuated Total Reflectance) sampling accessory (Smart iTR with diamond plate) by co-adding 32 scans in the 4000–400 cm^−1^ range at 4 cm^−1^ resolution.

Differential Scanning Calorimetry (DSC) measurements were carried out by a DSC Q2000 apparatus interfaced with a TA 5000 data station (TA Instruments, New Castle, DE, USA). The instrument was calibrated using ultrapure (99.999%) indium (m.p. = 156.6 °C; Δ*H* = 28.54 J g^−1^) as a standard. The calorimetric measurements were performed up to 300 °C at a heating rate of 5 K min^−1^ on samples amount of about 3–5 mg in open standard aluminum pans under nitrogen flow (45 mL·min^−1^).

Scanning Electron Microscopy (SEM) images were collected by a Zeiss Evo MA10 (Carl Zeiss, Oberkochen, Germany) microscope coupled with the Energy-Dispersive Spectroscopy (EDS) detector for microanalysis (X-max 50 mm, Oxford Instruments, Abingdon-on-Thames, UK). The samples for SEM analysis were sputtered with a thin layer of gold and analyzed at an acceleration voltage of the electron beam of 20 kV. The EDS data were obtained on loose powders.

N_2_ adsorption isotherms were obtained on powders, previously evacuated, with a Carlo ErbaSorptomatic1990 (Cornaredo, Milan, Italy). The data were analyzed with the Brunauer, Emmett, and Teller (BET) algorithm to determine the surface area. The pore size distribution was investigated by applying the Barret, Joyner, and Halenda (BJH) method on the desorption isotherms.

### 4.3. Pharmaceutical Measurements

#### 4.3.1. Drug Loading and Solubility

To determine drug loading, the hybrid compounds were placed in the fluid in which the Mlx is soluble (phosphate buffer pH 7.5) and left under stirring for a prolonged time until the readings of the drug concentration in solution became constant (UV detection, Lambda 25, Perkin-Elmer, Monza, Italy). To avoid the possibility of an overlap of the absorbance of the carrier with respect to the drug, the spectrum of the different molecules was previously acquired, and no overlap was found at the wavelength used.

Once the drug loading had been determined, the simple mixtures of the two components were prepared by weighing the corresponding quantities of the two compounds and mixing them in a Turbula apparatus (Bachofen Basel, Switzerland) for 15 min. The dissolution behavior of the physical mixtures, coded pmHAP-Mlx and pmHAP-Mg-Mlx, was compared with that of the two hybrids.

The shake-flask method [29] was used to determine the hybrids’ solubility in deionized water at 21 °C. An aliquot of the supernatant was taken and filtered through a 0.22 μm Millipore filter, the drug concentration was measured by UV detection, and the test was repeated until equilibrium was reached (three replicates).

#### 4.3.2. Dissolution Test

To simulate the in vivo condition of an oral administration, the dissolution tests were performed in some biorelevant fluids.

All samples were sieved on a 230 mesh grid, 63 μm, (Endecotts, London, UK).

The USP apparatus 2, paddle (Erweka DT-D6, Erweka GmbH, Dusseldorf, Germany) was used, at 37.0 ± 0.5 °C and 75 rpm (three replicates), in 900 mL of four different fluids: pH 7.5 phosphate buffer, as requested by the official monograph of the US Pharmacopoeia [30], of HCl 0.1 N, pH 1.0 (a condition that simulates the gastric environment in fasted conditions), pH 4.5 phosphate buffer (a condition that simulates the gastric environment in fed conditions) and deionized water. The dissolution media were prepared according to the reagent and buffer solutions section of the USP [31].

All the samples contain 7.5 mg of Mlx. The drug concentration was determined by UV detection (Lambda 25, Perkin-Elmer, Monza, Italy) and analyzed by the provided software (Winlab V6 software, Perkin-Elmer, Monza, Italy).

#### 4.3.3. Contact Angle

First, 100 mg of sample was placed in a cylindrical container of 1.5 cm in diameter and 3 mm in depth; then, the surface was smoothed with a blade. The images of a drop (10 µL) of the different fluids placed in contact with the surface of the different powder samples were acquired at progressive times (from t = 0 up to 300 s) using a Contact Angle Meter DMe-211Plus (NTG Nuova Tecnogalenica, Cernusco, Italy). The data were processed by the software supplied with the equipment (three replicates).

## 5. Conclusions

The meloxicam hybrids based on pure and Mg-doped hydroxyapatites were able to dramatically increase both the wettability and dissolution rate of the drug, which pertains to the BCS class II, i.e., drugs with low solubility and high permeability. The Mg doping reduced the crystallite sizes and the pore dimensions of HAP while increasing the surface area and pore volume. This can suggest that the drug could be mainly distributed on the HAP surface, favoring its subsequent release in the analyzed fluids. To increase the possible interest of the pharmaceutical market toward these hybrids, it is mandatory to increase the amount of adsorbed drug on hydroxyapatite. For the future, we plan to try other mixtures of solvents with also different ratios between them to make easier the drug dissolution and to increase the affinity toward hydroxyapatite, to test other kinds of doped hydroxyapatites, or to try other syntheses route to change morphology/particle dimensions of HAPs. However, the high efficacy of the organic–inorganic hybrids in improving the pharmaceutical characteristics of drugs was widely demonstrated in the paper, suggesting that they could become a pillar for the new pharmaceutical formulations.

## Figures and Tables

**Table 1:** The main refined structural parameters as derived from the Rietveld refinement are shown. The standard deviations are reported in the brackets. The calculated bond lengths together with the agreement indices Rwp and GoF are also reported.

	HAP	HAP-Mlx	HAP-Mg	HAP-Mg-Mlx
a, c (Å)	9.4237(2), 6.8823(1)	9.4232(1), 6.8806(2)	9.4206(3), 6.8642(2)	9.4285(5), 6.8643(2)
Cry size (nm)	22.8(1)	22.7(1)	13.6(2)	12.2(2)
Rwp/GoF	8.45/1.17	8.82/1.25	8.59/1.20	8.79/1.27
Atomic positions				
Ca1 (2/3, 1/3, z)	−0.0022(10)	−0.0016(9)	−0.0012(9)	−0.0019(8)
Ca2 (x, y, ¼)-Mg	−0.0135(12), 0.2444(15)	−0.0139(11), 0.2436(16)	−0.0152(10), 0.2421(13)	−0.0174(11), 0.2401(15)
P (x, y, ¼)	0.3749(5), 0.4006(8)	0.3757(3), 0.3993(2)	0.3763(4), 0.3993(2)	0.3791(3), 0.3947(1)
O1 (x, y, ¼)	0.4820(12), 0.3259(15)	0.4824(11), 0.3286(16)	0.4804(15), 0.3280(14)	0.4855(12), 0.3350(11)
O2 (x, y, ¼)	0.4660(10), 0.5908(14)	0.4669(12), 0.5922(11)	0.4610(13), 0.5859(14)	0.4658(16), 0.5833(14)
O3 (x, y, z)	0.2533(11), 0.3397(12), 0.0675(14)	0.2525(13), 0.3380(16), 0.0716(10)	0.2483(14), 0.3372(15), 0.0761(11)	0.2441(15), 0.3326(16), 0.0751(10)
O(H) (0, 0, ¼)	-	-	-	-
Bond lengths (Å)				
Ca1 Mean value	2.4244 (x3) 2.4336 (x3) 2.8176 (x3) 2.5585	2.4239 (x3) 2.4367 (x3) 2.8363 (x3) 2.5656	2.4201 (x3) 2.4428 (x3) 2.8375 (x3) 2.5668	2.4361 (x3) 2.4617 (x3) 2.8693 (x3) 2.5890
Ca2 Mean value	2.3046 (x2) 2.3636 2.3695 2.5392 (x2) 2.6292 2.4357	2.3267 (x2) 2.3640 2.3646 2.5236 (x2) 2.6455 2.4392	2.3529 (x2) 2.3560 2.4231 2.4831 (x2) 2.6281 2.4398	2.3360 (x2) 2.3501 2.4038 2.4758 (x2) 2.6744 2.4359
P Mean value	1.4912 1.5533 1.6001 (x2) 1.5612	1.4572 1.5749 1.5869 (x2) 1.5515	1.4393 1.5242 1.5863 (x2) 1.5340	1.3734 1.5424 1.6308 (x2) 1.5444

**Table 2:** Samples stoichiometry derived from EDS analysis. The data are an average of the results from different acquisitions in three points of interest. The acronyms ST and OBS mean stoichiometric and observed, respectively (see text).

	HAP	HAP-Mlx	HAP-Mg	HAP-Mg-Mlx
Ca/atom%	19.35	8.52	16.99	5.87
Mg/atom%	-	-	0.77	0.26
P/atom%	11.91	5.57	11.0	3.78
Ca/P(_ST_)	1.67	1.67	1.58	1.58
Ca/P(_OBS_)	1.62	1.53	1.54	1.55
Mg/Ca(_ST_)	-	-	0.053	0.053
Mg/Ca(_OBS_)	-	-	0.045	0.044

## Data Availability

Data available on request.

## Supplementary Materials

The following supporting information can be downloaded at https://www.mdpi.com/article/10.3390/molecules29112419/s1, Figure S1: XRD patterns of physical mixtures; Figure S2: Comparison between detailed XRD patterns; Figures S3 and S4: Rietveld refinements; Figure S5: Enlarged view of Figure 4C; Figure S6: EDS spectrum and maps of HAP-Mg sample; Figure S7: Solubility of Mlx, HAP-Mlx and HAP-Mg-Mlx in deionized water at 21 °C.
