# Supplementary material for: Physicochemical Characterization of Hydroxyapatite Hybrids with Meloxicam for Dissolution Rate Improvement
Source: Molecules. 2024 May 21;29(11):2419. doi: 10.3390/molecules29112419 (PMC11173451; doi:10.3390/molecules29112419)
Supplement: Supplementary file 1 [file molecules-29-02419-s001.zip › molecules-3004867-supplementary.pdf]

Physicochemical characterization of hydroxyapatite hybrids with Meloxicam for dissolution rate improvement

Lauretta Maggi, Valeria Friuli, Beatrice Cerea, Giovanna Bruni, Vittorio Berbenni, Marcella Bini

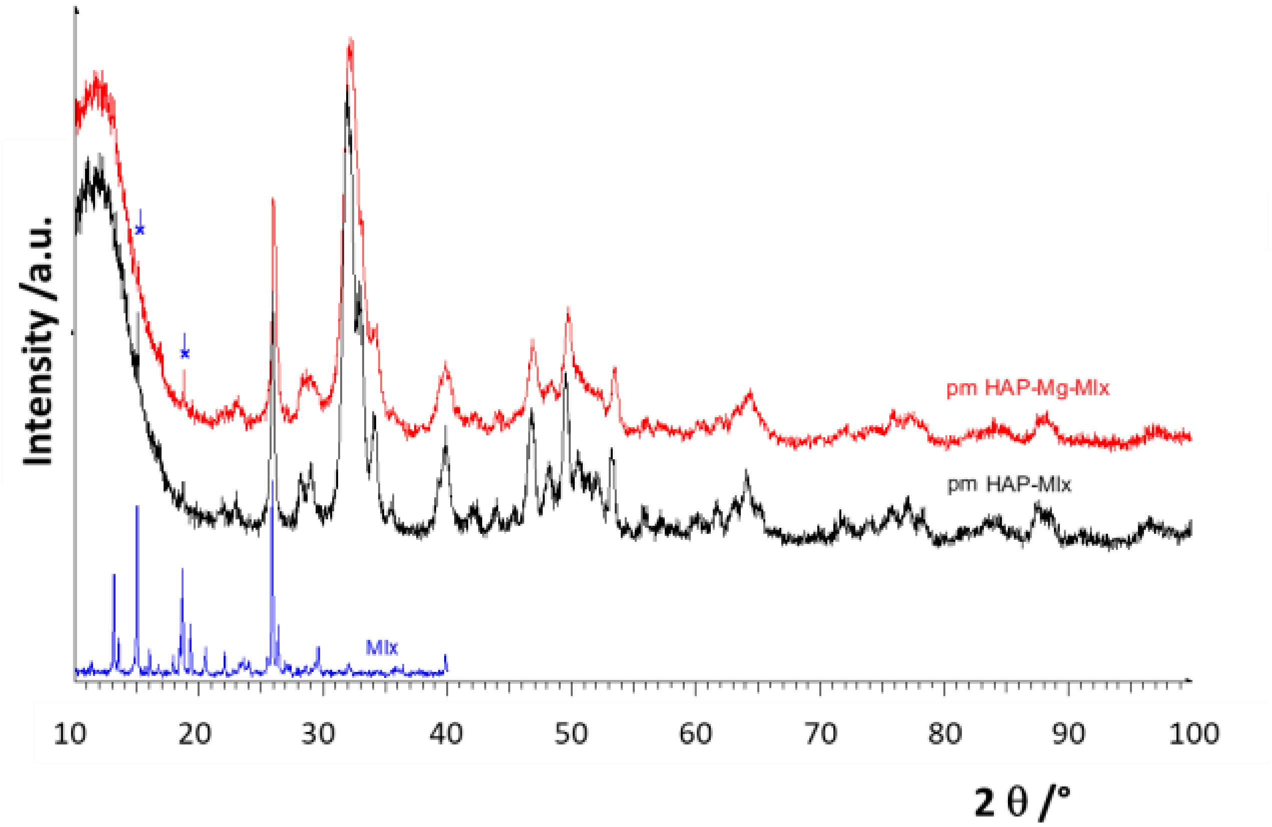

Figure S1 – XRD patterns of physical mixtures compared to that of pure drug. The cross mark the main peaks of meloxicam in the physical mixtures.

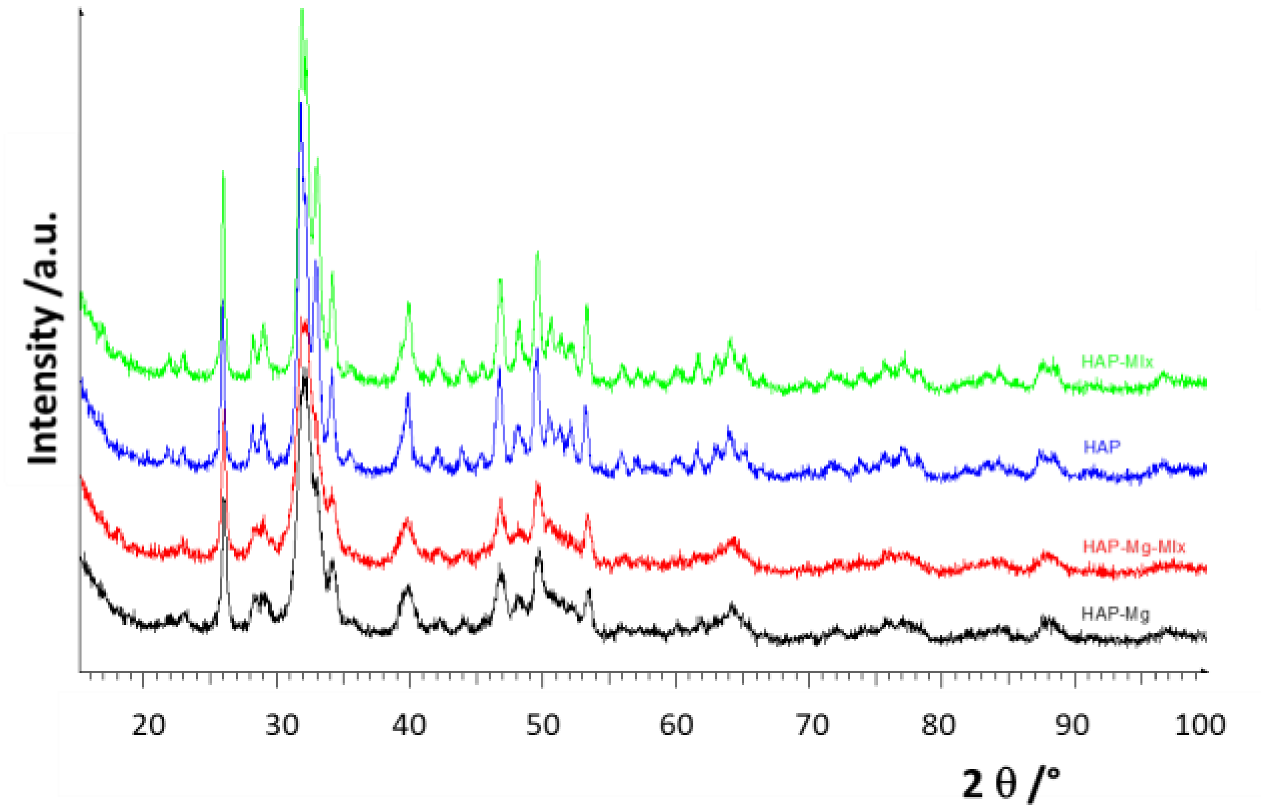

Figure S2 – Comparison between XRD patterns collected with a high time/step to perform the Rietveld structural refinements.

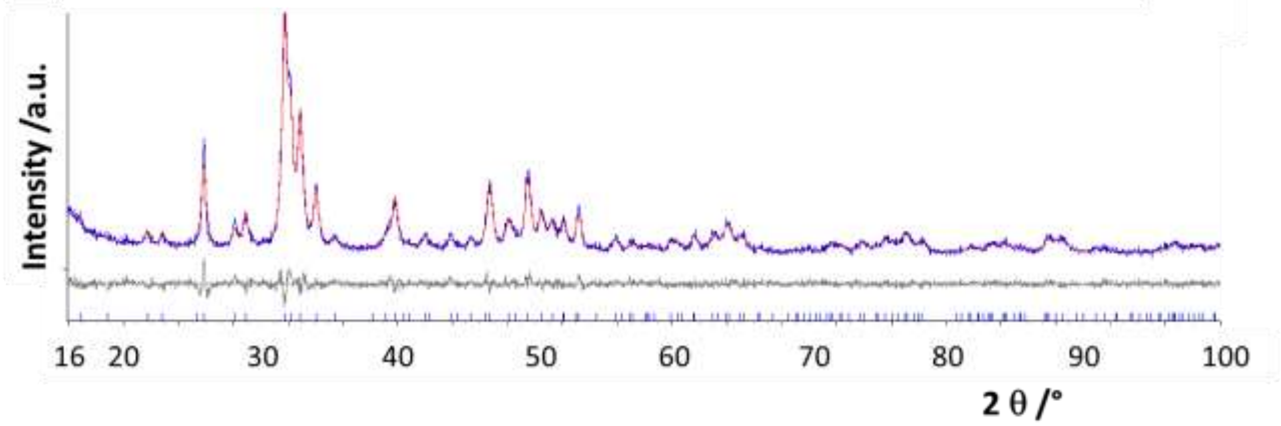

Figure S3 – Rietveld refinement results of HAP sample. The experimental pattern (blue) is compared with the calculated ones (red). In the bottom part, the difference curve (grey) and the bars of the expected peak positions are also shown.

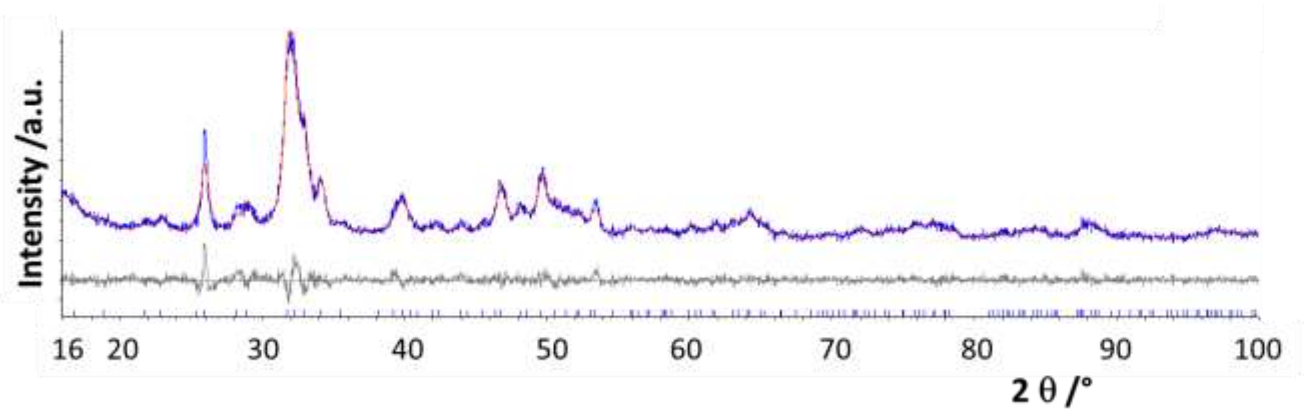

Figure S4 – Rietveld refinement results of HAP-Mg sample. The experimental pattern (blue) is compared with the calculated ones (red). In the bottom part, the difference curve (grey) and the bars of the expected peak positions are also shown.

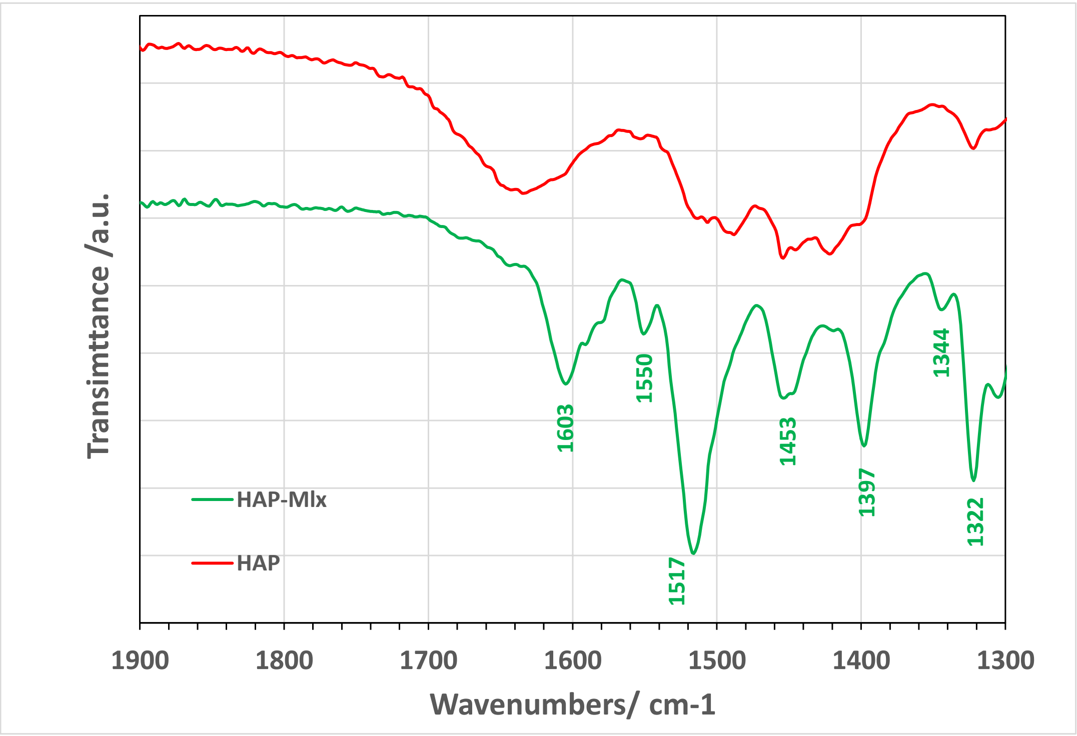

Figure S5 – Enlarged view of Fig. 4C to better evidence the Mlx peaks in the HAP-Mlx hybrid. The main peaks of drug are also labelled.

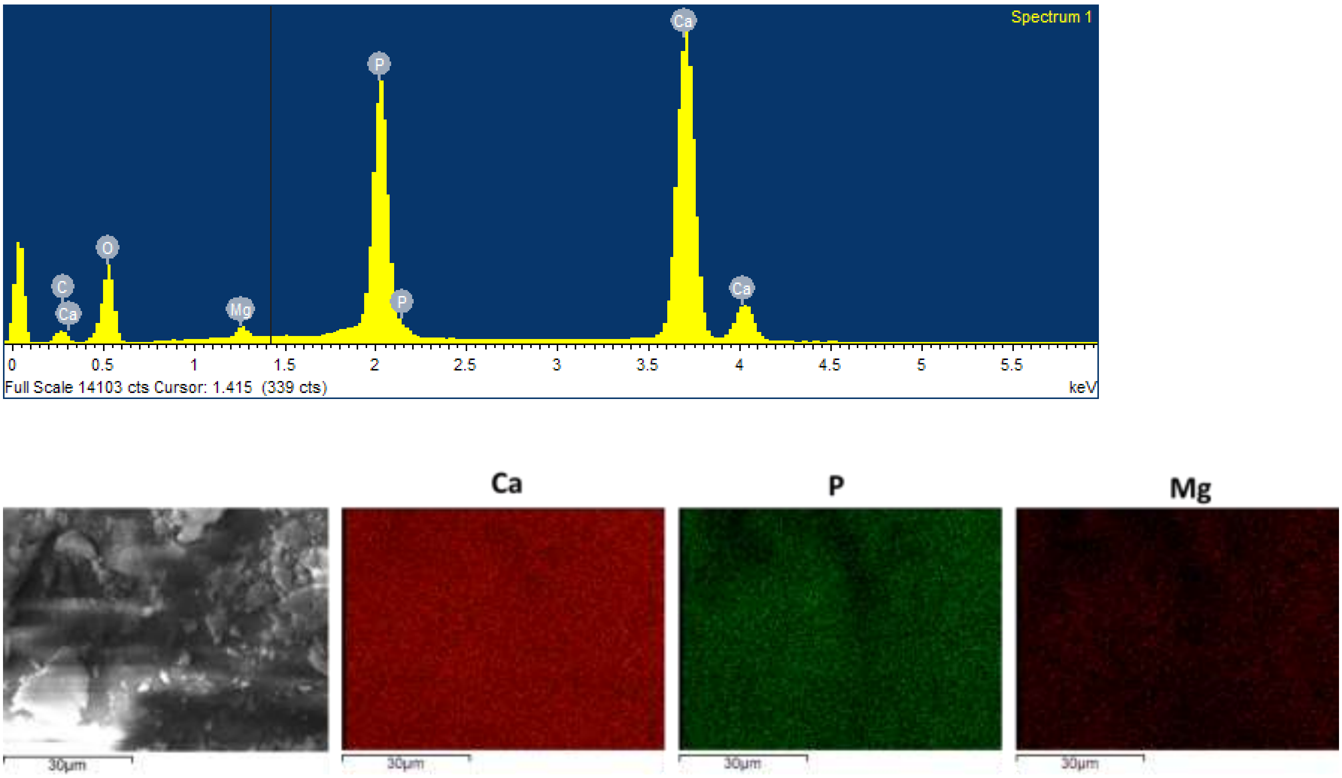

Figure S6 – EDS spectrum of HAP-Mg sample and SEM image with the corresponding maps of Ca, P and Mg elements.

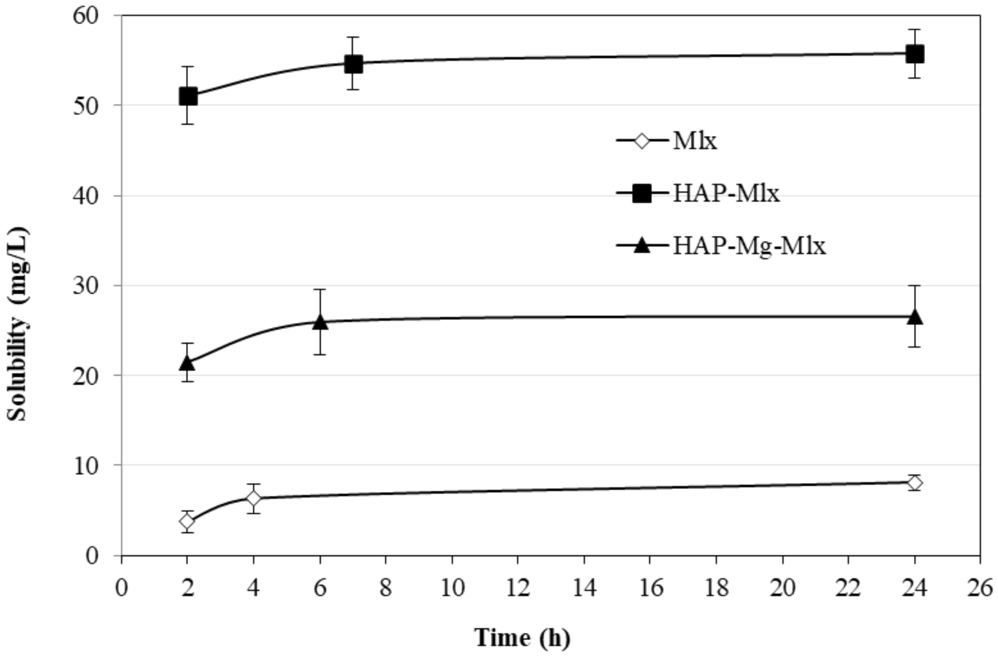

Figure S7 – Solubility of Mlx, HAP-Mlx and HAP-Mg-Mlx in deionized water at 21°C.
